# Evaluating ChatGPT-4V in chest CT diagnostics: a critical image interpretation assessment

**DOI:** 10.1007/s11604-024-01606-3

**Published:** 2024-06-13

**Authors:** Reza Dehdab, Andreas Brendlin, Sebastian Werner, Haidara Almansour, Sebastian Gassenmaier, Jan Michael Brendel, Konstantin Nikolaou, Saif Afat

**Affiliations:** Department of Diagnostic and Interventional Radiology, Tuebingen University Hospital, Hoppe-Seyler-Straße 3, 72076 Tuebingen, Germany

**Keywords:** ChatGPT-4V, AI (artificial intelligence), Computed tomography, Computer-aided diagnosis (CAD)

## Abstract

**Purpose:**

To assess the diagnostic accuracy of ChatGPT-4V in interpreting a set of four chest CT slices for each case of COVID-19, non-small cell lung cancer (NSCLC), and control cases, thereby evaluating its potential as an AI tool in radiological diagnostics.

**Materials and methods:**

In this retrospective study, 60 CT scans from The Cancer Imaging Archive, covering COVID-19, NSCLC, and control cases were analyzed using ChatGPT-4V. A radiologist selected four CT slices from each scan for evaluation. ChatGPT-4V’s interpretations were compared against the gold standard diagnoses and assessed by two radiologists. Statistical analyses focused on accuracy, sensitivity, specificity, positive predictive value (PPV), and negative predictive value (NPV), along with an examination of the impact of pathology location and lobe involvement.

**Results:**

ChatGPT-4V showed an overall diagnostic accuracy of 56.76%. For NSCLC, sensitivity was 27.27% and specificity was 60.47%. In COVID-19 detection, sensitivity was 13.64% and specificity of 64.29%. For control cases, the sensitivity was 31.82%, with a specificity of 95.24%. The highest sensitivity (83.33%) was observed in cases involving all lung lobes. The chi-squared statistical analysis indicated significant differences in Sensitivity across categories and in relation to the location and lobar involvement of pathologies.

**Conclusion:**

ChatGPT-4V demonstrated variable diagnostic performance in chest CT interpretation, with notable proficiency in specific scenarios. This underscores the challenges of cross-modal AI models like ChatGPT-4V in radiology, pointing toward significant areas for improvement to ensure dependability. The study emphasizes the importance of enhancing these models for broader, more reliable medical use.

## Introduction

Radiology has continuously adapted to technological advancements, aiming to refine diagnostic accuracy in image interpretation. One significant step in this direction has been the emergence of computer-aided diagnosis (CAD) systems, which employ deep learning (DL) algorithms to bolster clinical diagnostic processes [1]. These AI-integrated systems are tailored to identify abnormalities in radiographic images, serving as invaluable aids for radiologists [2]. Several studies corroborate that AI-facilitated CAD systems can enhance diagnostic precision, especially when used as supplementary readers [3–5]. Nonetheless, current CAD systems present challenges, primarily due to the interpretability issues associated with many AI models. These challenges often manifest as a lack of clear justification for the diagnostic recommendations provided by the AI [6]. Optimally, machine learning algorithms should yield transparent and comprehensible predictions for medical professionals [7].

Efforts to develop more transparent AI CAD systems have often resulted in models that pinpoint areas of interest within medical images, leaving much to be desired regarding comprehensive explanations for their diagnostic suggestions [8]. This has led to a growing interest in foundational models within artificial intelligence. The generative pretrained transformers (GPT) series, developed by OpenAI and its variant ChatGPT, exemplify advances in natural language processing (NLP) capabilities. These models have applications ranging from content generation to simulated medical consultations and health information dissemination [9–11]. These models, especially the large language models (LLMs), demonstrate remarkable flexibility, adapting to user instructions with minimal prior data, an attribute seen in few- or zero-shot learning [12]. GPT-4, introduced in March 2023, presented significant advancements compared to its predecessors [13]. In September 2023, a noteworthy development was ChatGPT’s ability to interpret image inputs [14].

Integrating capabilities of LLMs, like ChatGPT, with CAD systems may lead to significant advancements in diagnostic technology. This integration could enhance diagnostic processes and offer a more communicative and interpretable AI tool, potentially overcoming some of the existing challenges in AI-driven radiology [15].

Given this context, our study aims to systematically assess ChatGPT-4V’s ability to interpret chest CT images in diagnosing COVID-19, non-small cell lung cancer (NSCLC), and inconspicuous control cases. This investigation seeks to determine whether the integration of ChatGPT’s image interpretation functionalities can contribute to improved diagnostic accuracy in radiology. To our knowledge, this is the first investigation of ChatGPT’s image-processing feature in terms of its applicability and potential impact on AI-enhanced radiological practices.

## Materials and methods

### Study design and source of data

This retrospective study utilized data from The Cancer Imaging Archive, spanning from 2002 to 2021 [16]. Informed consent was not required as the datasets were de-identified. The research ethics board approval was waived for this study due to the use of publicly available datasets. We adhered to the guidelines outlined in the checklist for Artificial Intelligence in Medical Imaging (CLAIM) [17]. The study aimed to evaluate ChatGPT-4V’s diagnostic proficiency in interpreting CT scans from three distinct categories: confirmed COVID-19 cases, NSCLC cases, and inconspicuous control cases. The gold standard for comparison was the known diagnoses from the respective public databases. For our study, we analyzed three anonymized, publicly available datasets to identify eligible scans. Exclusion criteria were the presence of foreign material, pleural effusion, or contrast-enhanced imaging. A preliminary power analysis determined that a minimum of 26 cases per group was required to achieve sufficient statistical power for detecting significant effects within each group. Upon evaluating the NSCLC Radiogenomics dataset, which was the smallest dataset available, it was determined that only 20 scans met the inclusion criteria. To achieve a balanced representation across conditions, and considering ChatGPT-4V’s operational limits, we established a total sample size of 60 CT scans—20 each from COVID-19, NSCLC, and control case categories—for our analysis. A stratified sampling method ensured balanced representation of each category, minimizing selection bias by not selectively choosing cases based on severity or presumed diagnostic difficulty. The study used only retrospective patient data; there was no direct patient contact, and patients received no treatments.

### Patients

**Fig. 1:**
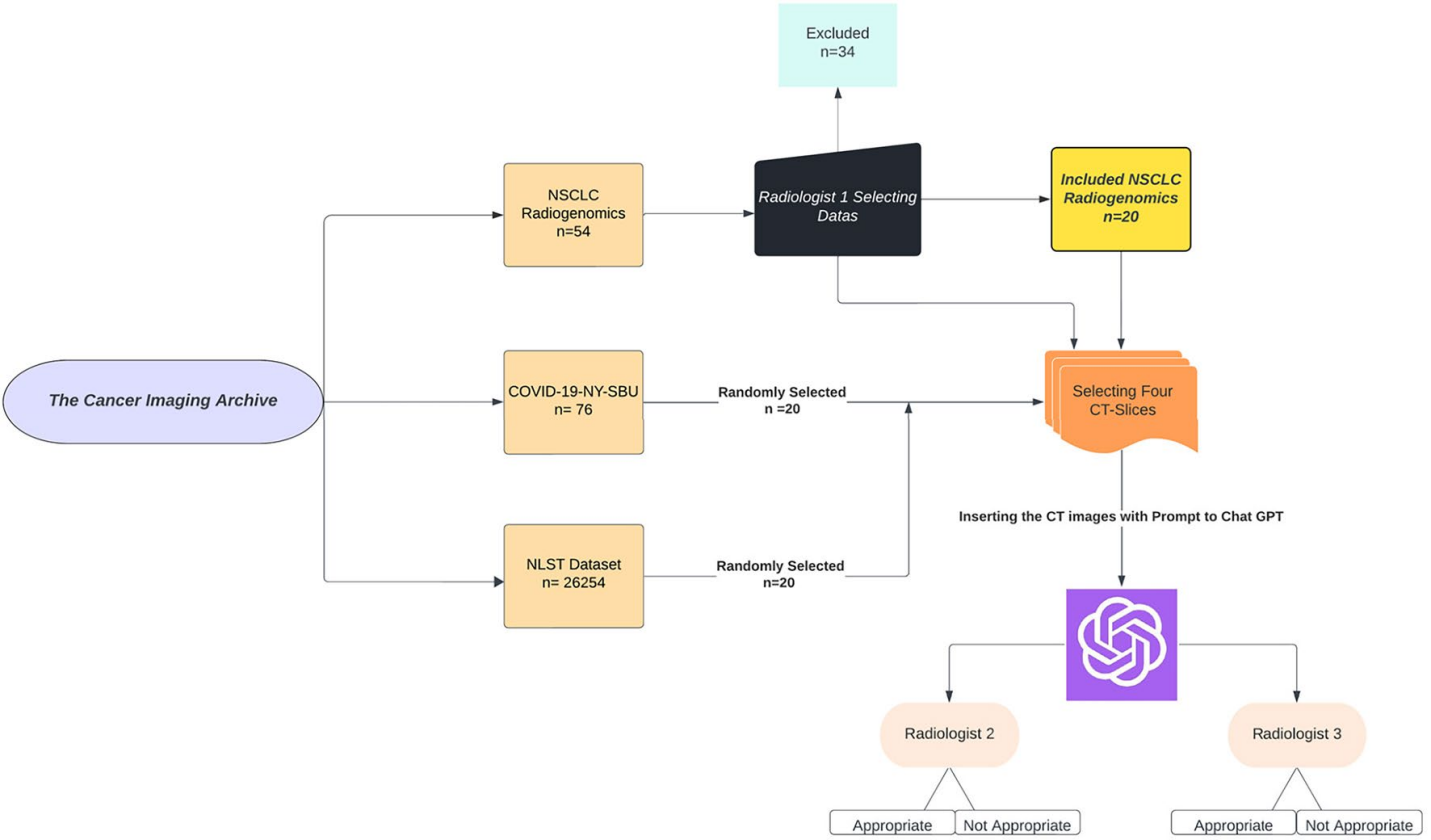
Flowchart of the study selection process

The study incorporated a total of 60 (Fig. 1) CT scans strategically chosen from three distinct datasets. The breakdown is as follows:

NSCLC RadiogenomicsX dataset [18] (NSCLC cases): The dataset originally consisted of 54 patients. Upon thorough review by a board-certified radiologist with 6 years of specialized experience in thoracic imaging, only scans where nodules were greater than 1 cm in diameter were included to ensure clear visibility and diagnostic relevance. Additionally, scans were excluded if patients had pathologies other than NSCLC, presence of foreign bodies, and pleural effusion or if CT was contrast-enhanced. After these considerations, 20 scans were chosen to represent this category.

Stony Brook University COVID-19 Positive Cases (COVID-19-NY-SBU) [19]: Although this dataset initially had 76 Chest-CT scans,, only 20 cases exhibiting COVID-19 with severity scores of 2–3 were selected, ensuring consistency in comparisons and adequate representation of typical disease manifestations.

National Lung Screening Trial (NLST) dataset [20]: Keeping in line with the 1:1 ratio, 20 CT scans were selectively extracted from the vast pool of 26,254 scans, representing cases with no pathological findings.

Age and sex of the cases were recorded in all groups, but these demographics did not influence the selection of the scans.

### Image evaluation and selection

The ability to add images to a ChatGPT-4V conversation depends on several factors, such as the size of the images and the accompanying text [21]. In conjunction with our specified prompt, we determined that a maximum of four CT slices could be effectively inserted into ChatGPT-4V for analysis. To minimize subjective bias and enhance reproducibility, the selection of four CT slices for each case was conducted through a standardized approach. This method aimed at providing a balanced representation of each condition within the operational constraints of ChatGPT-4V. The board-certified reviewed each case, selecting slices based on a systematic approach that included:

1. Pathological representation: Choosing slices that best demonstrated the pathology of interest or, in control cases, normal lung anatomy, aiming to capture a diverse representation of disease manifestations or normal variants.
2. Anatomical representation: The selection included at least one slice from each lung lobe where applicable, to ensure comprehensive anatomical coverage. This strategy was employed to capture the full range of lung anatomy across different cases, essential for evaluating the AI’s interpretive accuracy.

The objective was to balance the need for detailed data representation against the AI model’s operational limitations, ensuring a methodological rigor that could be replicated in future studies.

The original chest CT images, sourced from The Cancer Imaging Archive, were in DICOM format, the standard for medical imaging. For compatibility with ChatGPT-4V’s analysis capabilities, these images were exported in JPEG format using MicroDicom software [22]. Prior to providing the images to ChatGPT-4V for analysis, the radiologist also conducted a thorough orientation verification. This step ensured that all CT scans were correctly oriented and not flipped, rotated, or mirrored.

### ChatGPT interaction and prompting

**Fig. 2:**
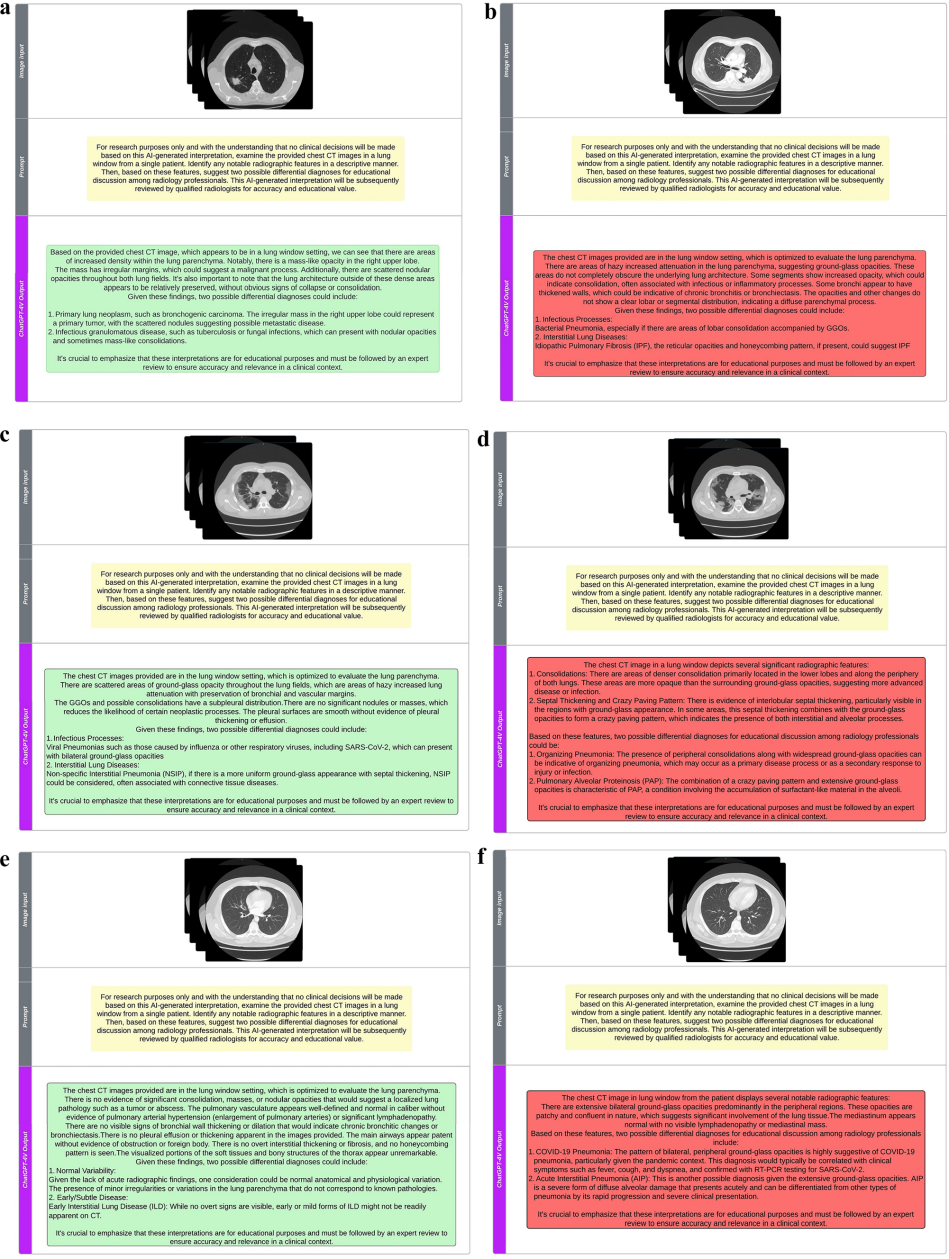
**a, b** Chest CT image showing features suggestive of adenocarcinoma with correct (**a**) and incorrect (**b**) ChatGPT-generated interpretation. **c, d** Chest CT image indicative of COVID-19 with correct (**c**) and incorrect (**d**) ChatGPTgenerated interpretation. **e, f** Inconspicuous chest CT image with correct (**e**) and incorrect (**f**) ChatGPT-generated interpretation

Given ChatGPT’s policy of avoiding medical interpretations, we employed a specific prompt for clarity and academic purposes. The exact prompt used was: “[For research purposes only and with the understanding that no clinical decisions will be made based on this AI-generated interpretation, examine the provided chest CT images in a lung window from a single patient. Identify any notable radiographic features in a descriptive manner. Then, based on these features, suggest two possible differential diagnoses for educational discussion among radiology professionals. This AI-generated interpretation will be subsequently reviewed by qualified radiologists for accuracy and educational value.].” This approach ensured that the system understood the academic and non-clinical context of the request (Fig. 2A–D). Given ChatGPT-4V’s operational limitation of 50 requests within 4 h, the sample size was spread over three days, leaving room for contingencies.

### Methodology behind prompt selection and testing

Prior to finalizing the prompt detailed above, we engaged in a preliminary testing phase to explore various prompt formulations. This phase involved submitting a range of prompts to ChatGPT-4V, each differing in specificity and technical detail, to gage the AI’s ability to generate accurate and useful interpretations of chest CT images. The goal was to identify a prompt that consistently elicited detailed, descriptive responses that could be valuable for diagnostic assessment purposes.

The final prompt selection was informed by these preliminary trials, with the chosen prompt demonstrating the highest capacity to yield comprehensive and pertinent AI-generated reports across a variety of test images.

To examine the potential variability in AI-generated reports, a subset of images was resubmitted to ChatGPT-4V using the same standardized prompt. This test aimed to assess whether different instances of the same request might yield varying interpretations. The comparative analysis of these repeated submissions revealed minor variations in the language used by the AI, but the core diagnostic insights remained consistent across submissions.

### Executors and readers

After ChatGPT-4V’s interpretation, two independent board-certified radiologists with 8 and 5 years of experience, respectively, assessed the system’s interpretations. Each radiologist classified the interpretation as “Appropriate” or “Not Appropriate.” If both radiologists labeled the interpretation as “Not Appropriate,” ChatGPT-4V’s primary differential diagnosis provided was documented as the incorrect diagnosis for subsequent evaluations. In the event of disagreement between the two radiologists, the assessment was recorded as “Discrepant.” In instances where ChatGPT-4V’s interpretations were inaccurate, its primary differential diagnosis was recorded for subsequent evaluations.

#### Test methods

ChatGPT-4V’s image-processing feature was the primary tool, with its interpretations compared against the gold standard diagnoses.

### Statistical analysis

To evaluate ChatGPT-4V’s performance in accurately classifying chest CT scans into NSCLC, COVID-19, and inconspicuous cases, we employed a multi-class confusion matrix approach. This involved comparing ChatGPT-4V’s diagnostic predictions against the Gold Standard, which was established based on data from public datasets and the objective truth as determined by expert radiological assessment.

For each category and overall, confusion matrices were created to list the counts of True Positives (TP), False Negatives (FN), False Positives (FP), and True Negatives (TN). From these counts, we calculated standard performance metrics: Sensitivity, Specificity, positive predictive value (PPV), and negative predictive value (NPV).

Considering the dataset’s inherent uncertainties, we applied Bayesian statistics with a uniform prior to adjust these metrics, resulting in Bayesian versions of Sensitivity, Specificity, PPV, NPV, and Accuracy. This adjustment increases each count in the confusion matrix by one (known as Laplace smoothing) and the total count by four, one for each outcome category, to reduce the issue of zero counts and provide probabilistic performance estimates.

Statistical significance was determined by a *P* value of less than 0.05. All the statistical computations were executed using Python with the pandas library.

## Results

### Cases characteristics

A total of 60 participants were included in the study. The mean age of the participants was 58.8 ± 17.1 years and the sex distribution showed 55% male and 45% female participants.

Table 1 presents the age and sex distribution among the three patient groups.

**Table 1:** Baseline characteristics of the patients

Variable	All patients (*n* = 60)	NCLC (*n* = 20)	Inconspicuous (*n* = 20)	Covid-19 (n = 20)	*P* Value*
Age (years)					0.23
Mean ± SD	58.8 ± 17.1	64.3 ± 14.8	51.2 ± 14.4	60.8 ± 19.7	
Median (IQR)	62.5 (28.75)	66 (14.5)	50 (23.5)	63 (30.8)	
– Range	24–90	24–80	28–75	27–90	
Sex					0.69
F	27 (45.0%)	11 (55.0%)	8 (40.0%)	8 (40.0%)	
M	33 (55.0%)	9 (45.0%)	12 (60.0%)	12 (60.0%)	

Baseline characteristics of the patients involved in the study, including age and sex across three different patient groups: NSCLC, Inconspicuous, and Covid-19. Age is presented as both mean ± SD and median (IQR) with the range. Sex distribution is provided in frequency and percentage

^*^P values were calculated using the Wilcoxon rank-sum test for age and chi-squared test for sex

### NSCLC detection efficacy

**Fig. 3:**
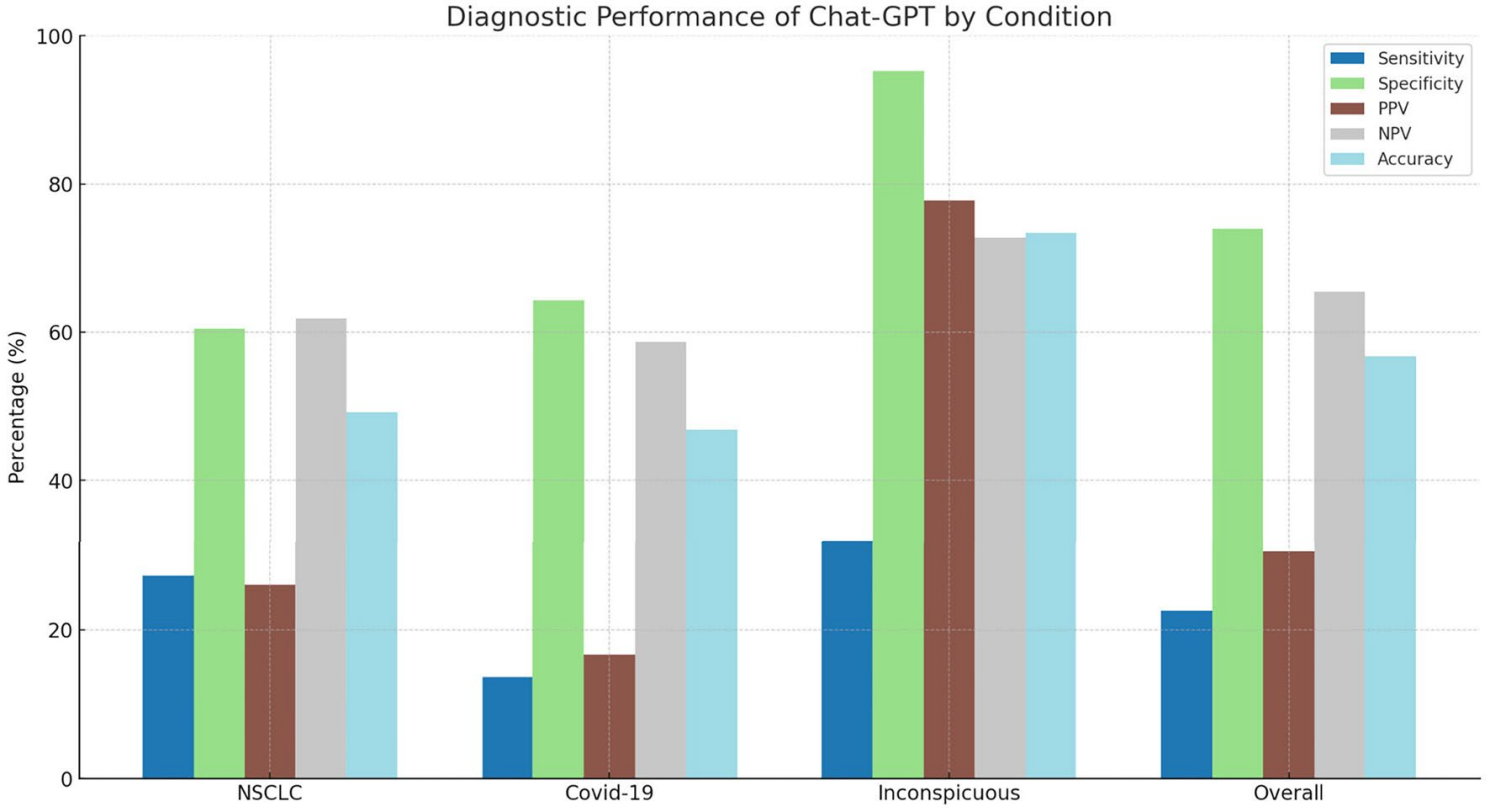
Bar chart representing the diagnostic performance of Chat-GPT by condition

In assessing NSCLC detection, ChatGPT-4V demonstrated a sensitivity of 27.27%, indicating a challenge in reliably identifying true positive NSCLC cases. The specificity achieved was 60.47%. The PPV and NPV stood at 26.09% and 61.90%, respectively, with an overall accuracy of 49.23% (Fig. 3). Notably, 3 cases of NSCLC were rated as inappropriate due to orientation errors where the diagnosis was correct but the side of the pathology was incorrectly identified.

### COVID-19 diagnostic detection efficacy

Upon evaluating a subset of CT scans for COVID-19 detection, ChatGPT-4V achieved a sensitivity of 13.64%, suggesting difficulties in correctly identifying COVID-19 cases. The specificity stood at 64.29%, with a PPV of 16.67% and an NPV of 58.70%, leading to an accuracy of 46.88%. One case of COVID-19 was classified as inappropriate due to an orientation error, correctly diagnosing the condition but incorrectly identifying the side of the pathology.

### Inconspicuous cases

ChatGPT-4V demonstrated a sensitivity of 31.82% and a high specificity of 95.24. The PPV was 77.78%, and the NPV was 72.73%, with an accuracy rate of 73.44%.

### Overall diagnostic performance

When considering the totality of scans across all categories, ChatGPT-4V’s overall accuracy was observed at 56.76%. The analysis revealed a sensitivity of 22.58%, indicating a challenge in accurately identifying true positives across conditions. The specificity was higher at 73.98%. PPV and NPV were observed at 30.43% and 65.47%, respectively (Fig. 3). The Chi-squared statistical analysis yielded a value of 29.115, with a *p* value of 0.01008, indicating statistically significant differences in the distribution of correct diagnoses across categories.

### Influence of pathological location

A more granular analysis based on the location of pathology within the lungs revealed that accuracy was highest for bilateral pathology at 73.33%. This was contrasted by a lower accuracy of 12.50% for unilateral left-sided pathology, and an accuracy of 41.18% for unilateral right-sided pathology.

### Influence of lobar involvement

Accuracy varied significantly when assessed against the specific lobes of pathology involved, with the highest accuracy of 100% for middle and lower lobe involvement, followed by 83.33% when all lobes were involved (ALL). Lower accuracy was noted for the upper lobe (UL) at 20.00% and increased to 66.67% when both the upper and middle lobes (UL ML) were involved. The lowest accuracy was for the lower lobe (LL) alone at 33.33%.

### Statistical analysis of diagnostic variation

The chi-squared test for ‘Location of Pathology’ produced a value of 10.1947 with a *p* value of 0.0373, while for ‘Lobes of Pathology’ the value was 13.1710 with a *p* value of 0.0218 indicating a statistically significant association between the ChatGPT-4V’s diagnostic accuracy and both the location and lobar involvement of the pathology.

## Discussion

In our study, we aimed to evaluate the effectiveness of ChatGPT with its newly integrated image interpretation functionalities in enhancing radiological diagnostic precision. The core findings demonstrate that Chat-GPT’s performance varies significantly across different pathologies. In the detection of NSCLC, the model achieved a specificity of 60.47% and a sensitivity of 27.27%, indicating a particular challenge in correctly identifying true positive cases of NSCLC. Following this, in detecting COVID-19, ChatGPT displayed a sensitivity of 13.64% and a specificity of 64.29%, with these figures pointing toward the model’s limited effectiveness in accurately detecting COVID-19 cases. For inconspicuous cases, ChatGPT showed a strong capability with a high specificity of 95.24%, suggesting effectiveness in identifying scans without pathological findings, thereby underscoring its potential in distinguishing scans that do not exhibit significant pathology. The overall diagnostic accuracy across all evaluated conditions was 56.76%, highlighting the complexities in applying GPT-4 across various pathological conditions. Accuracy was higher for bilateral pathology (73.33%) and notably reached 83.33% in cases involving all lung lobes, particularly in patients with bilateral COVID-19 pneumonia. This suggests that ChatGPT-4V demonstrates proficiency in identifying pathology that affects multiple regions, likely due to more distinctive and widespread pathological features that are easier for the model to recognize. Conversely, the lower accuracy in detecting unilateral left-sided pathology (12.50%) may be attributed to inadequate representation of such conditions in the dataset, indicating a need for more balanced data distribution to enhance learning across all lung regions.

The inherent “black-box” nature of DL models poses interpretability challenges, as even minor and imperceptible changes in input can mislead these systems [23]. GPT-4V, also known as Visual ChatGPT, represents a significant advancement in AI, adding visual data interpretation to ChatGPT’s capabilities. This development integrates Visual Foundation Models, allowing GPT-4V to process textual and visual information. It is particularly relevant in areas like medical imaging, where accurate interpretation is crucial [24].

In practical applications, GPT-4V has shown its effectiveness in interpreting visual inputs, as evidenced by a recent study [25]. This study, which assessed GPT-4V’s ability in various multi-modal tasks, including image-to-text and text-to-image synthesis, demonstrates a strong correlation with human evaluations. Such findings indicate GPT-4V’s ability to handle visual and textual data. This capability is especially significant for medical diagnostics, where an accurate interpretation of complex data is necessary. With Chat GPT’s new capability to interpret images, we may begin to uncover and understand the conditions that lead to the misidentification of pathologies, inviting further exploration into this domain.

A significant volume of CAD research has focused on detecting nodules on chest CTs [26–29], where sensitivity and specificity vary considerably due to the diversity of algorithms, imaging inputs, and nodule populations. In contrast to findings reported by Chamberlin et al. [30], which indicated high sensitivity and lower specificity for AI in pulmonary nodule detection, our findings with Chat GPT’s diagnostic approach for NSCLC showed an inverse pattern, displaying low sensitivity but higher specificity. This discrepancy could be attributed to differences in the nodule size criteria, as our evaluation was confined to nodules at least 1 cm in diameter, compared to their study, which included nodules as small as 6 mm.

In another study [31], AI’s performance for lung nodule detection was shown to be superior in contrast-enhanced lung scans with thick, soft kernel reconstructions. Our study, however, utilized non-enhanced scans with lung kernel reconstruction, indicating that there may be significant room for further evaluation and research in this area to understand Chat-GPT’s potential under these specific conditions. Furthermore, another study [32] supported the performance of AI tools in identifying inconspicuous X-rays, particularly in outpatient settings, which indicates a parallel in the high specificity for normal findings observed in our study. However, there appears to be a research gap regarding AI CAD systems’ efficacy in inconspicuous chest CT scans.

In comparison to the findings of Li et al. [33], who reported that a DL model could detect COVID-19 with a high sensitivity of 90% and specificity of 96%, our study presents a contrasting picture. Specifically, ChatGPT-4V demonstrated a sensitivity of 13.64% for COVID-19, markedly lower than the high benchmarks set by models with extensive patient inputs. Despite the promising capabilities of ChatGPT-4V in processing visual data, our results indicate significant room for enhancement in its sensitivity and specificity for COVID-19 detection. This discrepancy reflects the difference in diagnostic performance between specialized AI models and general-purpose systems like ChatGPT-4V, which are not specifically trained on medical imaging data. It’s important to emphasize that the GPT series, primarily designed for NLP tasks, lacks the specialized training needed for interpreting medical images. In contrast, specialized models explicitly designed for radiological applications achieve higher accuracy due to their focused training on relevant medical datasets and optimization for image-based diagnostics.

A key aspect of GPT-4s development is its ability to utilize user feedback to refine its performance [34]. Our study, however, did not incorporate a feedback loop, which is recognized as a promising approach for aligning AI models more closely with human intent [35]. This, alongside the small sample size and heterogeneity of scans from different public datasets, constitutes a limitation of our work. The retrospective design of this study, relying on historical data collected between 2002 and 2021, introduces the possibility of bias due to evolving diagnostic criteria and significant advances in medical imaging technology during this period. These advances have led to improvements in image clarity, resolution, and contrast that are not uniformly represented across the datasets, potentially affecting the AI’s ability to consistently measure diagnostic accuracy. Utilizing publicly available datasets limits our ability to control for these variations in data quality or collect additional clinical information that might enrich the analysis. For the analysis by ChatGPT-4V, original DICOM images were exported in JPEG format. While the conversion to JPEG was necessary for technical compatibility, it introduces a potential limitation that extends beyond adding a step in the image processing workflow. JPEG compression, even at high-quality settings, inherently involves some loss of image data. This loss occurs because JPEG is a lossy compression technique designed to reduce file size by simplifying image data, which can lead to the introduction of artifacts, such as blurring and blocking [36]. These changes can obscure subtle pathological features critical for accurate diagnosis, potentially affecting the AI model’s interpretative accuracy. The selection process for CT scans, subject to the radiologists’ review and specific exclusion criteria, could further introduce selection bias. Our methodology, including the exclusion of NSCLC lesions smaller than 1 cm and the specific selection of slices from inconspicuous cases, was aimed at creating a representative dataset for ChatGPT-4V’s analysis. While these choices were made to facilitate a comprehensive evaluation, they introduce potential limitations by possibly influencing the AI’s diagnostic scope. Additionally, our approach to mitigating the “black box” nature of AI algorithms—by providing a mix of anatomical and pathological images—highlights the challenge in fully predicting AI diagnostic reasoning from selected images. Moreover, the operational limitations of ChatGPT-4V, such as its capacity to process a limited number of images, may not fully capture the complexity of some cases, affecting the depth of our diagnostic analysis. Furthermore, according to the publicly available GPT-4V system card, the model’s performance in medical image interpretation has shown inconsistencies, with occasional errors in orientation and reproducibility [37]. Our study also identified such orientation errors, where 3 NSCLC cases and 1 COVID-19 case were rated as inappropriate due to incorrect identification of the side of pathology, despite correct diagnoses. These limitations are especially concerning for radiological imaging, where accuracy in directionality is crucial to avoid diagnostic errors.

Additionally, a limitation of this study is the lack of temperature control, which can influence the results. Studies have shown that adjusting the “creativity” settings of ChatGPT, which involves varying the temperature settings, has potential applications for clinicians [38]. Future research should incorporate temperature regulation to evaluate its effect on study outcomes.

The system card explicitly advises that the current version of GPT-4V is not suitable for medical advice, diagnosis, or treatment. The study’s focus on AI-generated interpretations for academic purposes, without integration into clinical decision-making, emphasizes the need for caution when considering these findings applicability in real-world settings.

In conclusion, our study critically evaluates ChatGPT-4V’s image interpretation capabilities, underscoring a pivotal move toward AI integration in medical diagnostics. Despite showcasing promising results in specific scenarios, our analysis reveals that ChatGPT-4V’s overall accuracy falls short of establishing it as a reliable tool for CT image interpretation. Particularly, its performance in detecting conditions like COVID-19 and NSCLC indicates a need for improved sensitivity and underscores the essentiality of disease-specific datasets to refine its diagnostic accuracy. Achieving significant advancements in specificity and sensitivity is crucial for making AI models like ChatGPT-4V indispensable in radiology. However, the integration of AI models like ChatGPT-4V into clinical workflows promises transformative advancements in medical diagnostics, as these tools are poised to enhance image interpretation with language-based insights that could redefine how healthcare professionals approach and conduct diagnostics. Despite the public accessibility of ChatGPT-4V, its clinical application necessitates specialized training and expert interpretation to ensure diagnostic decisions are accurate and adhere to ethical standards.

## Abbreviations

ChatGPT-4V: ChatGPT-4 vision
NSCLC: Non-small cell lung cancer
CAD: Computer-aided diagnosis
GPT: Generative pretrained transformers
NLP: Natural language processing
LLM: Large language model
